# Progressive Multifocal Leukoencephalopathy in a HIV Negative, Immunocompetent Patient

**DOI:** 10.1155/2016/7050613

**Published:** 2016-07-27

**Authors:** T. Nanda

**Affiliations:** Columbia College of Physicians and Surgeons, Columbia University Medical Center, 161 Fort Washington Avenue, New York, NY 10032, USA

## Abstract

Progressive multifocal leukoencephalopathy (PML) is a rare demyelinating disease most common in immunodeficient patients. It occurs due to reactivation of the John Cunningham Virus (JCV) and carries a poor prognosis, with a median life expectancy of 6 months. We report a case of a 66-year-old man with a history of HCV related cirrhosis (HCV) and hepatocellular carcinoma (HCC) who was found to have PML in the setting of a negative viral load in the CSF and a CD4+ >200. He initially presented with two weeks of mild confusion and word-finding difficulty concerning for hepatic encephalopathy. An MRI was notable for extensive T2/FLAIR hyperintensity signal in the left temporal lobe. Brain biopsy was positive for JCV. PML is rare in immunocompetent individuals, especially in the setting of a negative viral load. It is possible, however, that transient states of immunosuppression may have been responsible in this case. Although viral load was reported as negative, virus may still have been detected but was below the quantifiable threshold. It is important for clinicians to note that a negative result does not necessarily exclude the possibility of PML, and care should be taken to review lab values on viral load in closer detail.

## 1. Introduction

Progressive multifocal encephalopathy (PML) is a fatal demyelinating disease generally present in patients with severe immunosuppression such as HIV/AIDS, organ transplant recipients, those with hematological malignancy, and those receiving immunosuppressant therapy. PML, however, can also rarely present in immunocompetent patients, or in patients with transient or occult immunosuppression. Although limited to case reports, patients with a low to undetectable John Cunningham Virus (JCV) viral load may still present with PML. We present a patient with hepatic cirrhosis and hepatocellular carcinoma who was still found to have PML in the setting of a CD4+ count >200 and a negative viral load.

## 2. Case Presentation

A 66-year-old male with a past medical history of HCV related cirrhosis was admitted to the medicine service for altered mental status, worsening dyspnea on exertion, and paraphasic errors. His altered mental status had developed over the past two weeks and was characterized by mild confusion and word-finding difficulty. He had no other complaints and denied any recent travel or sick contacts.

On exam he was found to have some mild asterixis and word finding difficulty, often describing an object or concept rather than stating it (i.e., referring to staples as “wing things” or describing Clinton as the “future President's wife”). The rest of the exam was unremarkable with no other neurologic deficits. He was stating 97% on room air, which dropped to 92% with walking. The rest of his vitals were within normal range. Out of concern for hepatic encephalopathy he was treated with Lactulose and Rifaximin, with no improvement in mental status.

The rest of his workup was extensive. Infectious sources (blood, urine, CSF, and chest X-ray) were negative. Cardiac workup was unremarkable. An IR guided lumbar puncture showed four white blood cells with an 80% lymphocytic predominance. The rest of his CSF findings, including cytology and flow cytometry, were unremarkable. JCV viral load was reported as negative (<72 copies) by polymerase chain reaction assay. The patient had normal complement and was HIV negative, with negative ANA, paraneoplastic panel, NMO antibodies, ACE, myeloperoxidase antibodies, and serine protease 3. Of note, the patient's total WBC count was consistently low, ranging from 1.96 to 2.58, with an unremarkable differential.

In regard to his HCV, he had only partial response to PEG-Interferon + Ribavirin. A recent magnetic resonance cholangiopancreatography demonstrated malignant transformation of a dysplastic nodule, now 1.8 cm in size.

An MRI was also obtained for possible cerebral edema related to hepatic encephalopathy. Extensive T2/FLAIR hyperintensity was found in the left temporal lobe insula and subinsula region, left frontal horn, and left lateral ventricle. There were also multiple foci of T2/FLAIR hyperintensity in the left frontal and parietal lobe. The lesions did not show diffusion restriction and there was no hemosiderin on SWI sequences. The findings were thought to be characteristic of PML.

In the setting of a negative JCV viral load in the CSF, however, other etiologies were considered, including acute disseminated encephalomyelitis, focal seizures, vasculopathy, or other inflammatory causes such as sarcoidosis, although these were considered less likely. Primary neoplasm or metastatic disease was deemed unlikely, but per neurooncologic recommendations an MRI spectroscopy was performed which did not find increased blood flow or perfusion to the regions of T2/FLAIR hyperintensity on the prior MRI. Single voxel spectroscopy of the area with T2/FLAIR hyperintensity demonstrated elevated choline and lactate consistent with increased cell membrane turnover. The differential remained broad, consistent with either an active demyelinating process or a low-grade neoplasm.

At the time, due to a lack of diagnosis, the patient underwent a left temporal brain biopsy with superior temporal artery biopsy. Biopsy cytology was unrevealing, but final pathology returned positive for JCV by a combination of immunostaining, histopathologic features, and PCR, resulting in a final diagnosis of PML. To determine immune status, a T-cell panel was ordered twice, which showed consistently reduced total T cells (643, 512) and reduced helper T cells (342, 268), with a normal CD4+/CD8+ ratio.

## 3. Discussion

JCV occurs most often in childhood as an infection without apparent illness. Antibodies are present in eighty-six percent of adults [1]. It is a latent virus, resident in the kidneys and lymphoid organs. Reactivation of latent virus in the setting of severe immunosuppression results in the development of PML. JCV reactivation is a lytic infection of oligodendrocytes, consistent with our patient's spectroscopy findings of demyelination and increased cell membrane turnover. 79% of affected individuals have AIDS [2]. The rest are divided into patients with hematological malignancies, organ transplant recipients on immunosuppressive drugs, and most recently those receiving immunomodulating drugs such as Natalizumab, Rituximab, or Efalizumab for Crohn's disease, multiple sclerosis, and other dysimmune disorders [3–5]. PML has also been found in patients receiving HAART therapy for HIV, a condition called immune reconstitution inflammatory syndrome [6]. Prognosis is very poor, with a median survival in HIV negative patients of 3 months [7].

While classic PML presents in patients with <200 CD4+ cells and only in 5% of those with severe immunodeficiency [8], rare cases of PML in immunocompetent patients with and without underlying disease have been reported [9–11]. Those with underlying disease have been defined as “PML in the presence of occult or transient immunodepression,” presumably caused by conditions such as hepatic cirrhosis, chronic renal failure, pregnancy, dementia, and dermatomyositis. This makes the diagnosis of PML particularly challenging, as transient states of immunodepression or immunodysfunction may not always be apparent, and the number of additional conditions ranges from idiopathic CD4+ depression to solid tumor malignancies [12].

In our patient, the possible causes of his occult immunodepression are many and likely a combination of several factors. It is known that cell mediated response, particularly CD4+ cells, is integral to the containment of JCV. Even in situations where CD4+ and CD8+ levels are normal, failures of the immune response itself may be enough to allow the development of PML. In one study of 38 reported cases of PML in patients with possible occult or transient immunosuppression by Gheuens et al. [12], cirrhosis was found in seven PML patients. Three of those had documented CD4+ lymphocytopenia or leucopenia. It is also known that patients with HCV or HBV related liver disease have a marked depression in cell mediated immune function, especially in the setting of anemia and hypoalbuminemia [13]. Our patient had consistent leucopenia, anemia, and hypoalbuminemia (range 2.6 to 2.8) in the setting of HCV liver cirrhosis. There is also some evidence of solid malignancies causing immune dysregulation via tumor-derived soluble factors such as IL-10 and TGF-beta. It is possible that patient's newly malignant liver nodule played a contributing role [14].

It is also possible that patient's immune dysregulation is idiopathic in nature. Idiopathic CD4+ lymphocytopenia (ICL) is defined as a CD4+ count <300 or a CD4+ count <20% of the total T cell count on two occasions, with no evidence of HIV infection, defined immunodeficiency, or immunosuppressing therapy [15]. Our patient meets all criteria, aside from a T-cell panel from two separate occasions, as he was lost to followup after discharge. ICL is a heterogeneous disease with a still unknown pathophysiology. Some studies suggest its development after an opportunistic infection, such as cryptococcosis or extrapulmonary tuberculosis [16]. Increased CD4+ cell apoptosis and a reduction in IL-7 receptors have also been implicated [17]. Bone marrow studies of patients with ICL have shown a reduction in early CD4+ stem cell precursors [18]. It is unknown if our patient ever had an opportunistic infection that may have predisposed him to developing ICL. In addition, it is difficult to determine if his immunodeficiency can instead be defined by HCC or HCV cirrhosis, as these are competing causative factors.

It is likely that an earlier and more extensive immune constitution workup would have been pursued had less emphasis been placed on the reportedly negative JCV viral load found in his CSF [9]. Prior cases of PML in patients with low JCV viral load have been reported, as low viral load does not correlate with the extent of disease [19]. PCR detection of JCV DNA in the CSF has been shown to have a specificity of 92% to 100% and a sensitivity of 74% to 92% when compared to brain biopsy for patients with HIV-associated PML [20]. With a 100% specificity, it is presumed that any level of virus in the CSF should be concerning for PML, with a range from undetectable to 7.71 log copies/mL found in patients with PML in a prior study by Bossolasco et al. [21].

## 4. Conclusion

In this case, the interpretation of a negative JCV viral load in the CSF may have contributed to a delay in diagnosis. This value was reported as negative due to the inability to quantify viral load when less than 72 copies. This does not exclude the presence of virus, however. Considering the evidence of PML in patients with low to undetectable JCV titers, any level of detection should spark clinical suspicion. It is also important to consider occult or transient immunosuppressive states even with a CD4+ count >200, as this may not accurately reflect the patient's true immune status. In patients with suspected PML and no immunodeficiency, taking a detailed history on prior opportunistic infections may add to a possible diagnosis of ICL. The purpose of this discussion was to highlight the many pitfalls in evaluating a complicated neurological story for JCV reactivation, especially considering the varied and transient immunodepressive states associated with cancer or chronic disease.
